# Identification of candidate host serum and saliva biomarkers for a better diagnosis of active and latent tuberculosis infection

**DOI:** 10.1371/journal.pone.0235859

**Published:** 2020-07-20

**Authors:** Olivia Estévez, Luis Anibarro, Elina Garet, Ángeles Pallares, Alberto Pena, Carlos Villaverde, Víctor del Campo, África González-Fernández

**Affiliations:** 1 Immunology Group, CINBIO, Centro de Investigaciones Biomédicas, Universidade de Vigo, Vigo, Spain; 2 Instituto de Investigación Sanitaria Galicia Sur (IIS-GS), Vigo, Spain; 3 Grupo de Estudio de Infecciones por Micobacterias (GEIM), Spanish Society of Infectious Diseases (SEIMC), Spain; 4 Tuberculosis Unit, Department of Infectious Diseases and Internal Medicine, Complejo Hospitalario Universitario de Pontevedra, Pontevedra, Spain; 5 Department of Microbiology, Complejo Hospitalario Universitario de Pontevedra, Pontevedra, Spain; 6 Epidemiology Unit, Alvaro Cunqueiro University Hospital, Vigo, Spain; Institut de Pharmacologie et de Biologie Structurale, FRANCE

## Abstract

In our work, we aim to identify new candidate host biomarkers to discriminate between active TB patients (n = 28), latent infection (LTBI; n = 27) and uninfected (NoTBI; n = 42) individuals. For that, active TB patients and their contacts were recruited that donated serum and saliva samples. A multiplex assay was performed to study the concentration of different cytokines, chemokines and growth factors. Proteins with significant differences between groups were selected and logistic regression and the area under the ROC curve (AUC) was used to assess the diagnostic accuracy. The best marker combinations that discriminate active TB from NoTBI contacts were [IP-10 + IL-7] in serum and [Fractalkine + IP-10 + IL-1α + VEGF] in saliva. Best discrimination between active TB and LTBI was achieved using [IP-10 + BCA-1] in serum (AUC = 0.83) and IP-10 in saliva (*p* = 0.0007; AUC = 0.78). The levels of TNFα (*p* = 0.003; AUC = 0.73) in serum and the combination of [Fractalkine+IL-12p40] (AUC = 0.83) in saliva, were able to differentiate between NoTBI and LTBI contacts. In conclusion, different individual and combined protein markers could help to discriminate between active TB and both uninfected and latently-infected contacts. The most promising ones include [IP-10 + IL-7], [IP-10 + BCA-1] and TNFα in serum and [Fractalkine + IP-10 + IL-1α + VEGF], IP-10 and [Fractalkine+IL-12p40] in saliva.

## Introduction

Tuberculosis (TB) remains one of the major causes of deaths worldwide, being responsible of 1.5 million deaths in the year 2018 [1]. An accurate TB diagnosis and treatment of people infected with *Mycobacterium tuberculosis* (*Mtb*) is key to prevent the death of millions of people every year. However, there are still great limitations in TB diagnostics.

Active TB diagnosis by detection of acid-fast bacilli in sputum has low sensitivity [2] and sputum culture has a long turnaround time until the final result; *ex vivo M*. *tuberculosis* gene amplification test (GeneXpert MTB/ RIF) provides rapid results with high sensitivity [3]. However, this is a relatively expensive test that requires specialized infrastructure not always available in low-income areas [4]. Furthermore, none of these tests allows the detection of latent TB infection (LTBI) [5]. Currently, there is not a gold standard test for the detection of LTBI, hence the Tuberculin Skin Test (TST) or the Interferon-gamma Release Assay (IGRA) are used for this purpose. Both these tests detect immunological memory against *Mtb* antigens and neither of them can discriminate between active TB and LTBI [6].

Considering all the limitations mentioned above, it is evident the necessity of new diagnostic tools that allow the discrimination between active TB patients, latent TB infection and uninfected individuals. Direct *ex vivo* assays that could be adapted to affordable point-of-care testing are desirable. In addition, these tests should use easy-to-access biological specimens that can be obtained from all individuals. Serum and saliva samples pose several qualities that make them attractive candidates for this purpose. Serum samples are easy to collect by specialized personnel and require minimum sample processing [7]. However, one of its limitations is its invasiveness and the requirement of skilled technicians for collection. Saliva samples, on the other hand, are non-invasive, cost-effective, easy to store and easy to obtain by non-specialized personnel [8,9]. In addition, saliva represents a mucosal sample connected with the respiratory tract, the main infection route of *Mtb*. For this reason, we believe that saliva could not only be a potential tool for TB diagnosis but also a source of information of the events that take place at the mucosal level, which is of great importance in the context of pulmonary TB.

Identification of host biomarkers in serum and saliva that help to discriminate between groups is key for the development of improved diagnostic tools. Previous investigations have been done using serum, plasma [10–19] and saliva [16,20,21] samples, that identified some candidate TB biomarkers. However, there is still a lack of consensus of which are the most powerful ones to differentiate active and latent TB infection or between healthy people with or without infection. For this reason, we have performed a study in which we have analysed some of the markers most broadly studied in the context of tuberculosis, such as IFNγ or the IFN-inducible protein 10 (IP-10), but also other cytokines, chemokines and growth factors that could be contributing to the infection, and therefore present a distinct signature on the different study groups. Our research provides information of new candidate biomarkers in both serum and saliva samples, that could help on improving the diagnostic tools available to date.

## Material and methods

### Study participants

Participants included in the current study were recruited within the framework of the H2020 project “Eliciting Mucosal Immunity to Tuberculosis” (EMI-TB; H2020-EU.3.1: Societal Challenges; “Health, demographic change and well-being”. Reference 643558). The recruitment was conducted between September 2015 and July 2017 in at the Tuberculosis Unit in the “Complexo Hospitalario Universitario de Pontevedra” (Galicia, North-West of Spain).

The study included microbiologically-confirmed active pulmonary TB patients and their contacts, classified as uninfected (NoTBI) or with latent tuberculosis infection (LTBI). Participants were eligible for the study if they were willing to give written informed consent. People were excluded from the study if they were under 18 years old; they were pregnant; had received anti-TB treatment before; a TST was conducted in the last 3 months before the recruitment; were co-infected with HIV or were under any immunosuppressive treatment including inhaled corticosteroids. We also excluded people with diabetes, end-stage renal disease, alcoholism or any autoimmune disorder and, in general, any other immunosuppressive state as considered by the attending physician. In addition, contacts matching any of the following conditions were also excluded: a previous TB diagnosis or TST/IGRA documented; the presence of an old-healed lesion on chest on chest radiography; a recent (<3 months) vaccination with live-attenuated strains and having had any other active infection during the previous month. The study was approved by the Galician Ethics Committee (registry number: 2014/492) and written informed consent was obtained from all participants.

### Diagnostic tests

Diagnosis of TB contacts was based on the TST and, when indicated, with an IGRA test, following the Spanish consensus for TB diagnosis [22].

TST was carried out following the Mantoux method using two units of tuberculin RT-23 (PPD, Statens Serum Institute, Copenhagen, Denmark). After 48–72 hours, the induration diameter was measured. Participants with ≥ 5 mm induration area were considered TST positive. The Quantiferon^TM^-TB Gold In-Tube Kit (Cellestis Ltd, Carnegie, Australia) was used to measure the Interferon gamma production in antigen-stimulated blood T cells after incubation with the antigens ESAT-6, CFP10 and TB7.7, following the manufacturer’s instructions. The cut-off value for a positive IGRA result was 0.35 IU/mL. A “window period” was considered in contacts with a negative TST/IGRA in their first visit. In these cases, a second test was conducted 8–10 weeks after the last possible exposure to the index case [23].

Patients with active pulmonary TB were all microbiologically confirmed by means of culture and/or Nucleic-Acid Amplification in respiratory specimens. Contacts of TB patients with positive TST/IGRA that did not show any symptoms and had no evidence of clinical and radiological disease were finally diagnosed with Latent TB infection.

### Sample collection and processing

A total of 97 participants were recruited including 28 active TB patients, 42 uninfected contacts and 27 LTBI contacts. All individuals donated 10 mL of peripheral venous blood collected in SST II *Advance* (Vacutainer, BD; Plymouth, UK) serum separator tubes. The tubes were centrifuged at 1300 g for 10 min at room temperature and the serum fraction was collected, aliquoted and kept at -80°C until their use.

Saliva samples were collected in 15 mL polypropylene tubes up to a volume of 7–10 mL per participant and kept at 4°C during sample processing. The tubes were centrifuged at 300 g for 5 min and the supernatant was collected and treated with a protease inhibitor (Complete Tablet Mini, Roche; Mannheim, Germany) to avoid protein degradation. Saliva supernatants were de-contaminated by mechanical disruption on a BeadBeater device (Mini BeadBeater-16, BioSpec Products; Bartlesville, OK, USA) applying three pulses of agitation of 20 s in the presence of 0.1 mm zirconia beads (BioSpec Products; Bartlesville, OK, USA). The supernatant was recovered after centrifugation and filtered through a 0.22 μm cell strainer. Processed saliva samples were kept at -80°C and gradually thawed on ice on the day of the assay.

### Multiplex immunoassay

Customized Milliplex kits (modified from the “*Human Cytokine/Chemokine Panel I*” (HCYTOMAG-60K), “*Human Cytokine/Chemokine Panel II*” (HCYP2MAG-62K) and “*Human Th17*” (HTH17MAG-14K) panels) were used to evaluate different proteins of the immune response. Several protein markers were studied in serum and saliva samples, including interferon (IFN)-γ and -α2, Tumor necrosis factor (TNF)-α and -β, IL-1α, IL-1β, IL-2, IL-3, IL-7, IL-12p40, IL-12p70, IL-15, IL-16, IL-17A IL-17F, IL-21, IL-22, IL-23, IL-32, IL-1Ra, IL-4, IL-5, IL-9, IL-10, IL-13, IL-6, IL-27, IL-33, the soluble form of the CD40 ligand (sCD40L), Transforming growth factor (TGF)-α, IL-8 (CXCL-8), B cell-attracting chemokine 1 (BCA-1 or CXCL-13), Eotaxin (CCL-11), Fibroblast Growth Factor-2 (FGF-2), FMS-related tyrosine kinase 3 Ligand (FLT-3L), Fractalkine (CX3CL1), GRO (CXCL1), interferon-inducible protein 10 (IP-10 or CXCL-10), Monocyte chemoattractant protein 1 (MCP-1 or CCL-2) and 3 (MCP-3 or CCL-7), Macrophage-derived chemokine (MDC or CCL-22), Macrophage inflammatory protein (MIP)-α and -β (or CCL-3 and CCL-4, respectively), RANTES (CCL5), Epidermal growth factor (EGF), Granulocyte-colony stimulating factor (G-CSF), Granulocyte-macrophage colony-stimulating factor (GM-CSF), Platelet-derived growth factor (PDGF)-AA and -AB/BB and the vascular endothelial growth factor (VEGF). Serum samples were used without further dilutions after thawing and saliva samples were diluted ½ in sterile PBS. Serum and saliva samples were evaluated on separate plates. Samples from all three groups were included on the same plate. Samples were analysed using a MagPix device (Luminex; Austin, Texas, USA) with the xPonent 4.2 software. The quality control samples for all analytes were within the expected range. A calibration curve was built based on the concentration and median fluorescence intensity of the standards that were used to calculate the concentration of each sample.

### Statistical analysis

Differences between groups were evaluated using the Kruskal-Wallis test to look for general differences between the three groups (NoTBI, LTBI and TB) followed by a Dunn’s Test corrected for multiple comparisons. Differences with a *p*-value ≤ 0.05 were considered significant. The diagnostic accuracy of the markers with significant differences between groups was assessed by the receiver operator characteristics (ROC) curve analysis. The cut-off values for each parameter were determined by the highest Youden Index [24] to maximize the sensitivity and specificity of the test. Individual markers with an area under the ROC curve (AUC) >0.7 were used to study the classification abilities of marker combinations. Binary logistic regression was applied to calculate the predicted probability of combined biomarkers for discrimination between every two groups. The predicted probability was used to construct the ROC curves and calculate the AUC. The diagnostic efficacy of the marker combinations and individual markers were compared based on the differences of the respective AUCs using the DeLong method. Statistical analysis and graph representation were performed using GraphPad Prism version 6.00 for Windows (GraphPad Software; CA, USA), IBM SPSS version 23 for windows (SPSS Inc., Chicago, Ill., USA) and the free software R (version 3.4.3).

## Results

A total of 97 patients were recruited: 28 microbiologically confirmed active TB patients, 42 uninfected contacts and 27 LTBI contacts. Table 1 summarizes the study participants, regarding age, sex and previous BCG vaccination, with a higher proportion of males, especially in the TB group.

**Table 1:** Characteristics of study participants.

	All	NoTBI	LTBI	TB
**Number of cases**	97	42	27	28
**Age** mean (range)	43 (19–76)	40 (19–76)	48 (19–71)	41 (21–72)
**Females/Males**	38/59	22/20	11/16	5/23
**BCG** naive/vaccinated	73/24	29/13	20/7	24/4

### Screening of host markers in serum samples

An initial screening was performed analysing the concentration of 46 different proteins in serum of 49 participants from the three groups. Among them, 18 markers (IL-21, IL-27, BCA-1, EGF, FGF-2, Eotaxin, TGFγ, IFNγ, GRO, MDC, IL-17A IL-8, IP-10, MCP-1, MIP-1γ, MIP-1β, TNFγ and VEGF) were detected in 90–100% of the samples, two proteins were detected in 70–90% of the samples (GM-CSF and IL-7), five in 60–70% of them (IL-17, G-CSF, IL-1Ra, IL-1β and IL-6) and the remaining were below the minimum detectable level in more than half of the samples, hence they were not considered for further analysis. Among those with detectable levels in most of the samples, we applied a Kruskal-Wallis test and only six (IL-6, IL-7, IP-10, TGFα, TNFα and BCA-1) presented significant (p < 0.05) differences between groups or a trend (p < 0.1) that suggested a different distribution. These marker candidates were then analysed in the rest of the participants and the differences between groups were further investigated.

### Individual host markers in serum

**Fig 1:**
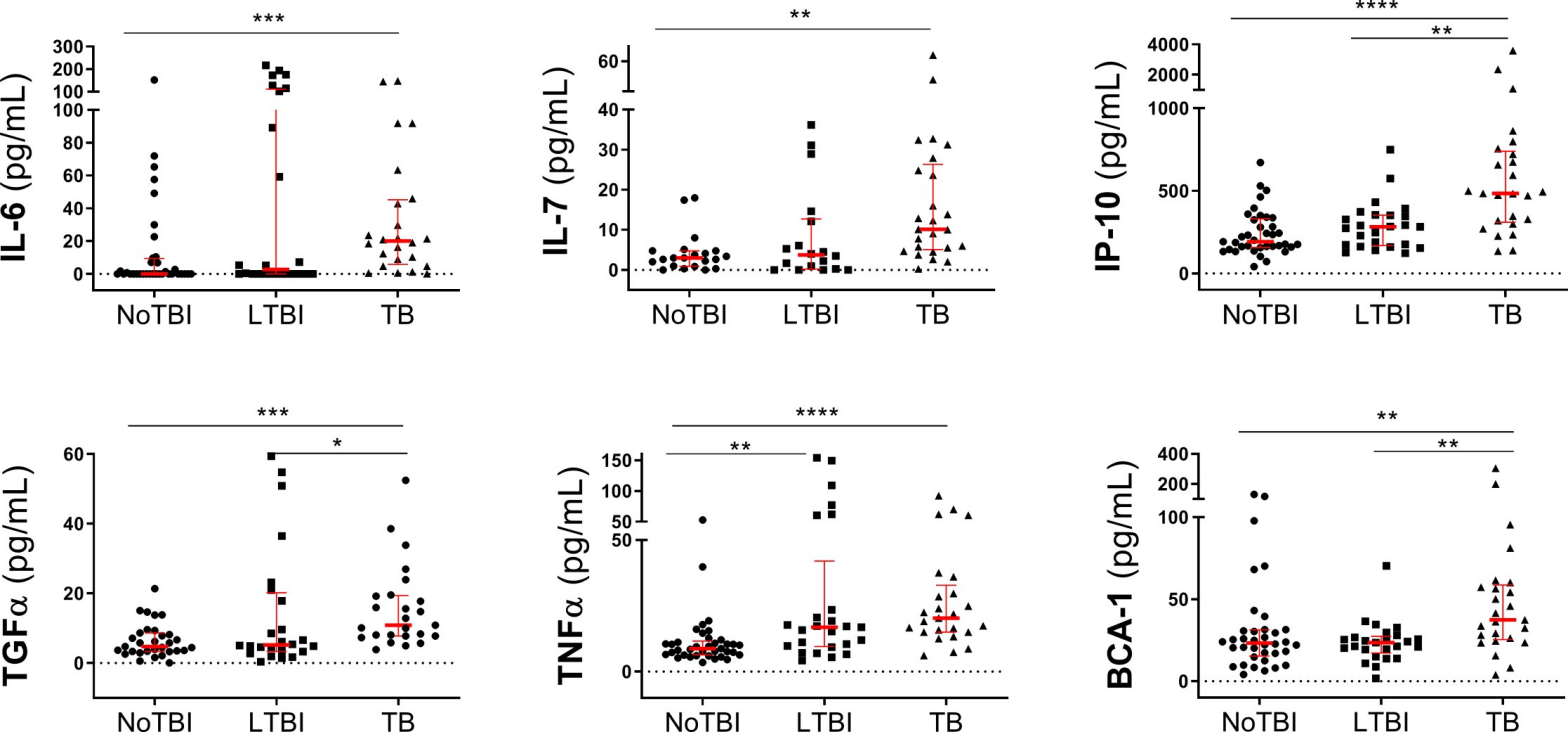
Concentration of protein markers detected in serum samples. The horizontal bars represent the median and the interquartile range. Differences between study groups were calculated using a Kruskal-Wallis test followed by a Dunn´s test corrected for multiple comparisons. Significant differences between groups are expressed as: * p < 0.05; ** p < 0.01; *** p < 0.001; ****p < 0.0001.

The baseline concentration of the selected cytokines including IL-6, IL-7, IP-10, TGFα, TNFα and BCA-1 showed greater concentrations in serum from active TB patients than in their uninfected contacts (*p* values ranging between 0.05–0.0001) and, in the case of IP-10, TGFα and BCA-1, also compared to LTBI contacts (p < 0.05 –p < 0.01). No major differences were found between the two contact groups, except in TNFα, where LTBI contacts showed significantly higher concentration of this cytokine than in the NoTBI contacts (p < 0.005) (Fig 1, Table 2 and S1 Table). In all cases, TB patients showed higher concentrations of the studied cytokines, but LTBI contacts showed intermediate levels between uninfected contacts and TB patients.

**Table 2:** Median levels and interquartile ranges of selected candidate host markers detected in serum samples from TB patients and contacts.

Marker	NoTBI	LTBI	TB
**IL-6**	0 (0–9.38)	2.74 (0–111.5)	20 (5.74–45.14)
**IL-7**	3.00 (0.91–4.78)	3.74 (0.19–12.73)	10.12 (5.08–26.36)
**IP-10**	191.6 (157.6–329.9)	282 (167.7–352.9)	483.5 (309.1–738.6)
**TGFα**	4.73 (3.36–8.62)	5.179 (3.36–20.18)	10.84 (7.76–19.38)
**TNFα**	8.74 (6.3–11.57)	16.8 (9.53–42.01)	20.32 (15.02–32.79)
**BCA-1**	23.26 (15.28–31.26)	23.56 (17.15–27.36)	37.4 (25.37–58.73)

**NoTBI**: Uninfected contacts; **LTBI**: Contacts with latent TB infection; **TB**: Active Tuberculosis patients.

A ROC curve analysis was conducted to assess the diagnostic accuracies of individual host markers performing pair-wise comparisons. This analysis showed that the six markers present promising results (AUC > 0.7) differentiating active TB and NoTBI contacts, being TNFα, IL-7 and IP-10 the ones with the best discriminatory capacity (S1 Table).

For discrimination between LTBI and active TB patients, the best three protein markers were IL-7, IP-10 and BCA-1, while for NoTBI and LTBI contacts, only TNFα showed significant statistical differences (S1 Table).

### Combination of host markers in serum

These individual host markers that showed significant differences between groups were further explored trying different combinations based on binary logistic regression. The best combination using the minimum number of markers was selected using a backward stepwise selection. For discrimination between active TB and NoTBI contacts, the combination with the highest AUC (0.87) and accuracy (80.1%) was IP10 + IL7 (Table 3), which presented a better performance than the individual cytokines, although no significant differences (S1 Table) were found (Z = 1.0926, *p-*value = 0.2746 and Z = 1.5859, *p-*value = 0.1128 respectively). The inclusion of either TNFα and/or TGFα (both with an AUC > 0.8), did not improve the combination IP10 +IL17.

**Table 3:** Accuracy of marker combinations in serum for the diagnosis of TB infection.

	Combination	AUC	Acc.	Sens. (%)	Spec. (%)
**NoTBI *vs* TB**	IP10 + IL7*	0.88	80.85	**80**	**81.82**
** **	IP10 + IL7 + TNFα	0.88	78.72	**80**	77.27
** **	IP10 + IL7 + TGFα	0.88	80.85	**80**	**81.82**
** **	IP10 + IL7 + TNFα + TGFα	0.88	78.72	**80**	77.27
**LTBI *vs* TB**	IP10 + BCA1	0.83	80.00	72	**88.00**
** **	IP10 + BCA1 + IL7	0.79	70.45	72	68.42
**NoTBI *vs* LTBI**	TNFα **	0.73	-	60	**83.78**

**AUC**: area under ROC curve. **Acc**: Accuracy. **Sens**: Sensitivity; **Spec**: Specificity.

*Selection made by backward elimination (Likelihood ratio).

**In the case of NoTBI versus LTBI, only TNFα showed differences.

For discrimination between LTBI and active TB patients, the three markers IP-10, BCA-1 and IL-7, that individually showed a good performance, were combined using logistic regression. The combination of IP10 + BCA-1 (the two with highest AUC) showed an AUC of 0.83 with 88% specificity and 72% sensitivity (Table 3). This combination also showed a better performance than these two markers alone. Adding up IL-7, however, did not improve the results.

In the case of the comparison between NoTBI and LTBI contacts, we only found differences in the level of TNFα (Table 3 and S1 Table), so combinations with other cytokines were not studied.

In summary, the study in serum of just 4 cytokines: IP-10, IL-7, BCA-1 and TNFα could help to identify the three TB populations. Increasing levels of IP-10 + IL-7 would allow the discrimination between active TB and NoTBI; IP-10 + BCA-1 from active TB and LTBI contacts, and only TNFα for differentiation between NoTBI and LTBI contacts.

### Screening of host markers in saliva samples

An initial screening of potential host markers in saliva samples was performed analysing 44 different proteins in 66 participants. Six out of the 44 candidates (IL-23, IL-33, IL-15, IL-17A, IL-2 and IL-3) were undetectable in all samples and six (Eotaxin, IL-4, IL-5, IL-9, sCD40L and TNF-β) were only detected in < 15% of the saliva samples. All of them were excluded for further analysis. On the other hand, 18 protein markers (EGF, Fractalkine, GMCSF, GRO, GCSF, IL-1α, IL-1β, IL-1Ra, IL-7, IL-8, IFN-α2, MCP-1, MDC, PDGF-AA, PDGF-AB/BB, TGF-α, TNF-α and VEGF) were above the minimum detectable level in >90% samples; Nine (IL-16, FGF-2, FLT-3L, IL-13, IL-6, IL-9, IFN-γ, IP-10 and MIP-α) were detected in 70–90% of the samples and six (IL-10, IL-12p40, IL-12p70, MCP-3, MIP-β and RANTES) had detectable levels in 50–70% of the samples. From them, thirteen markers (IL-16, EGF, Fractalkine, GRO, IL-12p40, IL-1α, IL-6, IFN-α2, IP-10, MCP-1, MIP-alpha, TGF-α, VEGF) showed significant differences between groups (p<0.05) when applying a Kruskal-Wallis test. These were selected for further analysis using the remaining saliva samples.

### Individual host markers in saliva

**Fig 2:**
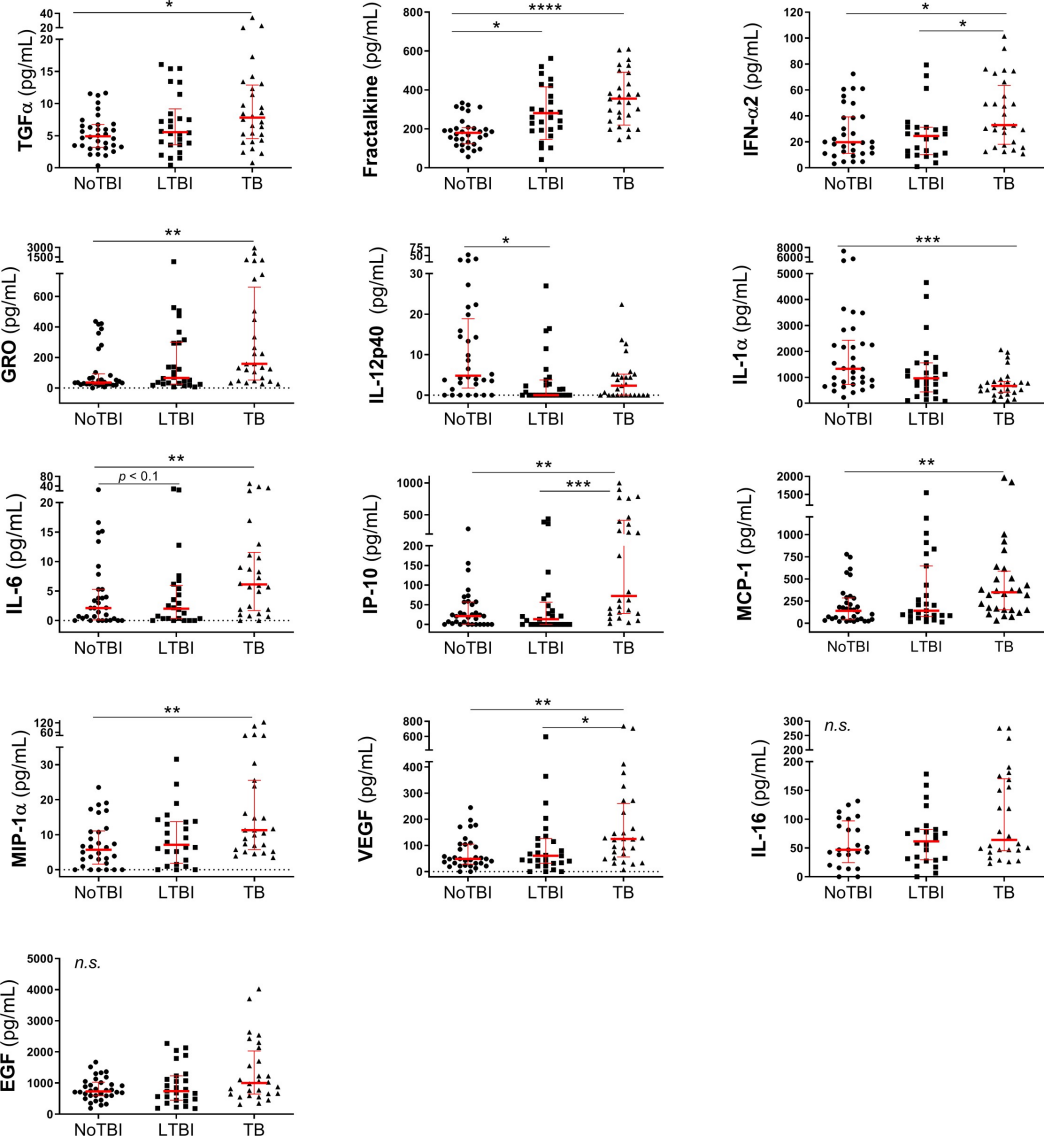
Concentration of protein markers detected in saliva samples. The horizontal bars represent the median and the interquartile range. Differences between study groups were calculated using a Kruskal-Wallis test followed by a Dunn´s test corrected for multiple comparisons. Significant differences between groups are expressed as: * p < 0.05; ** p < 0.01; *** p < 0.001; ****p < 0.0001 and ns: no significant differences.

The thirteen candidate markers selected in saliva samples (see material and methods) were studied in a total of 89 participants from the three groups (34 NoTBI, 27 LTBI and 28 TB). This analysis showed that, overall, these protein markers were present in higher concentrations in saliva of active TB patients, with the exception of IL-12p40 and IL-1α, that were more abundant in saliva of NoTBI contacts (Table 4, Fig 2). In most cases LTBI contacts showed an intermediate level between NoTBI and TB patients.

**Table 4:** Median levels and interquartile ranges of selected candidate host markers detected in saliva samples from the TB patients and uninfected and with latent infection contacts.

Marker	Median (interquartile range)		
	NoTBI	LTBI	TB
**TGFα**	4.92 (3.20–6.74)	5.56 (3.59–9.18)	7.822 (4.53–12.89)
**Fractalkine**	179.4 (123.7–206.4)	258.8 (192.3–381.4)	346.1 (244.4–474.6)
**IFNα2**	19.7 (11.15–39.24)	24.55 (10.16–31.24)	32.91 (18.1–63.53)
**GRO**	35.77 (24.87–92.34)	65.38 (25.69–300.6)	158.1 (51.72–661.4)
**IL-12p40**	4.807 (1.76–18.88)	0 (0–3.756)	2.326 (0–5.2)
**IL-1α**	1331 (726.8–2430)	965 (436.2–1559)	670.5 (415.6–840.5)
**IL-6**	2.1 (0.11–5.27)	2.0 (0.32–5.97)	6.1 (1.68–11.56)
**IP-10**	21.1 (0–57.04)	13.05 (0–56.35)	72.49 (27.6–415.3)
**MCP-1**	140.5 (43.23–285)	141.9 (75.96–647.2)	349.8 (152.8–589.3)
**MIP-1α**	5.719 (1.6–11.1)	7.123 (1.851–13.77)	11.32 (5.766–25.55)
**VEGF**	48.57 (27.79–104.9)	60.28 (31.76–126.4)	124.7 (56.14–260.3)

**NoTBI**: Uninfected contacts; **LTBI**: contacts with latent infection: **TB**: Active Tuberculosis patients; **AUC**: Area under the ROC curve; **Cut off**: marker concentration cut off with the best Youden index; **CI**: Confidence interval.

The greatest differences were found between active TB and NoTBI contacts, with most of the markers (TGF-α, Fractalkine, IFN-α2, GRO, IL-6, IP-10, MCP-1, MIP-1a and VEGF) showing significant differences (*p* < 0.05 –*p* < 0.0001) between these two groups, except for IL-12p40, which only showed significant differences between NoTBI and LTBI (*p* < 0.05), and IL-16 and EGF, that did not present significant differences between groups. In addition, IFN-α2 (*p* < 0.05), IP-10 (*p* < 0.001) and VEGF (*p* < 0.05) were significantly higher in active TB compared to LTBI contacts and Fractalkine was also significantly higher (*p* < 0.05) in LTBI than in NoTBI contacts (Fig 2).

The discriminatory capacity of each marker with significant differences between groups was evaluated performing a ROC analysis (S2 Table). The most promising host marker for discrimination between active TB and NoTBI in saliva was Fractalkine, with an AUC of 0.87, 78.57% sensitivity and 81.25% specificity at the selected cut-off (S2 Table). Other markers with good performance (AUC > 0.7) were GRO, IL-1α, IP-10, MCP-1, MIP-1α and VEGF (S2 Table).

As for the markers that were able to discriminate between LTBI and active TB, we found that IFN-α2 and IP-10 were the most promising ones (S2 Table). The sensitivity and specificity values were between 58.62% and 80.77%, showing a slightly worse performance than markers from serum samples. Finally, Fractalkine (AUC = 0.73; 69.2% Sensitivity and 75% Specificity) and IL-12p40 (AUC = 0.73; 74.1% Sensitivity and 72% Specificity) showed the best performance discriminating between NoTBI and LTBI contacts (S2 Table).

### Combination of host markers in saliva

Combinations of individual candidate markers in saliva were studied including those with an individual AUC >0.7. Candidate markers were fitted into logistic regression models to evaluate their classification performance. For discrimination between active TB and NoTBI, the combination of Fractalkine + IP10 + IL1α + VEGF was selected using a backward elimination approach, which showed an AUC of 0.88 (83.61% accurately classified samples, 91.2% specificity and 74% sensitivity) (Table 5). The combination presented a significantly better performance than individual IP-10 (Z = 1.9167, *p*-value = 0.05), IL1α (Z = 2.1925, *p*-value = 0.02834) or VEGF (Z = 6.6681, *p*-value = 2.591e-11), and despite differences with Fractalkine were not significant (Z = 1.1516, *p*-value = 0.2495), it did show a higher AUC than this chemokine alone.

**Table 5:** Accuracy of marker combinations in saliva for the diagnosis of TB infection.

	Combination	AUC	Acc.	Sens. (%)	Spec. (%)
**NoTBI *vs* TB**	Fractalkine + IP10 + IL1a + VEGF*	0.88	83.61	74.07	91.18
**LTBI *vs* TB**	IP10 + IFNα2	0.68	59.62	51.85	68.00
	GRO + IL-6 + IP10 + MIP1α*	0.77	65.22	64.00	66.67
**NoTBI *vs* LTBI**	Fractalkine + IL-12p40	0.83	82.76	76.92	87.50

*Selections made by backward elimination (likelihood ratio). **AUC**: area under the ROC curve. **Acc**: Accuracy. **Sens**: Sensitivity; **Spec**: Specificity.

Two different combinations were assessed to discriminate between LTBI and active TB. First, we tried combining the candidates with the highest individual performance (IP-10 and IFNα2), which showed an AUC of 0.67 (59.6% accuracy, 68% specificity and 51.9% sensitivity). A second combination was assessed using a backward elimination approach. The selected combination (GRO + IL6 + IP-10 + MIP1α) showed a better performance, with an AUC of 0.77. The differences between the ROC curves of these two combinations, however, were not significantly different, nor did they improve the individual performance of IP-10 and IFNα2 (Table 5).

Finally, a marker combination for discrimination between NoTBI and LTBI contacts was assessed combining Fractalkine and IL-12p40 (Table 5). This combination showed a better performance than the two markers alone, with an AUC of 0.83, and a ROC curve significantly better than that of Fractalkine (Z = 2.7566, *p*-value = 0.005841) and improved trend compared to IL-12p40 alone (Z = 1.6817, *p*-value = 0.09262).

In summary, different markers in saliva showed promising results: Fractalkine + IP10 + IL1α + VEGF discriminate between active TB and NoTBI contacts; Fractalkine + IL-12p40 differentiate LTBI from NoTBI contacts. On the other hand, discrimination between LTBI and TB using a marker combination did not improve the performance of individual IP-10 or IFNα2.

## Discussion

New tools for rapid and accurate TB diagnosis are needed in order to identify all *Mtb* infected people and differentiate active and latent TB infection. Saliva and serum samples, which are relatively easy to obtain, are ideal specimens for the identification of candidate biomarkers that could be used for the development of the required diagnostic tools. In our work, we analysed up to 50 different markers in serum and saliva and identified six markers in serum (IL-6, IL-7, IP-10, TGFα, TNFα and BCA-1) and nine in saliva (Fractalkine, GRO, IL-1α, IP-10, MCP-1, MIP-1α, VEGF, IFNα2 and IL-12p40) that showed promising potential for that purpose.

As we showed here, the host markers that best define either the serum or saliva signature are different for each specimen, even though the initial screening included almost the same array of markers. These results evidence the different composition of serum and saliva. Although saliva content can include components derived from blood, these analytes usually present different concentrations in both fluids [25]. This explains why, in our work, some proteins can be a promising biomarker in one specimen (such as Fractalkine in saliva) and yet be below the minimum detectable concentration in a high proportion of serum samples. In fact, previous investigations comparing serum and saliva samples in the context of TB infection [14,16] reported different concentrations of the same host proteins in both specimens. This supports the approach followed in the present study, where an independent analysis and marker selection was made for serum and saliva.

Among the host candidates identified in serum samples, IP-10 was the one with the best performance differentiating active TB patients from both uninfected and LTBI contacts, as proved by its area under the ROC curve. These results support the research done by other groups [26–30], confirming the suitability of this marker for TB diagnosis. As opposed to TB diagnostic tests based on the IFNγ concentrations (IGRA), which require an incubation period with *Mtb* antigens, IP-10 has been shown in ours and other studies [26,31–33] to be increased in serum, plasma and urine of TB patients without requiring further stimulation. Of note, in our work IFNγ was rejected for further analysis in the preliminary screening, as it did not show significant differences between groups. Hence indicating a poor utility of IFNγ as TB biomarker without prior *in vitro* stimulation.

Moreover, we showed that accuracy of IP-10 as an individual biomarker can be improved when combined with IL-7 for discrimination between active TB and NoTBI contacts, or combined with BCA-1 to differentiate active TB and LTBI. Although previous studies have analysed an array of biomarkers in serum or plasma samples for TB diagnosis [10–16], none of them have proposed IL-7 or BCA-1 as potential biomarkers before. Our results suggest that they could not only be used in combination with IP-10, but they showed a good performance differentiating active TB from both NoTBI and LTBI contacts when used individually, based on their respective AUCs.

Biologically speaking, a higher concentration of IP-10 and BCA-1 in serum samples from active TB patients could be related with immune cell migration to the site of infection. IP-10 and BCA-1 are two chemokines with chemoattractant properties over cells expressing the receptor CXCR3 [34,35], which include Th1 cells, one of the major participants in cellular immunity against *M*. *tuberculosis* infection [36]. IL-7, on the other hand, has been associated with enhanced recall responses of *Mtb*-specific CD4^+^ T cells *in vitro* [37], therefore suggesting a protective role. However, other studies described an impaired T-cell sensitivity to IL-7, with decreased soluble and membrane-bound IL-7 receptor and increased IL-7 plasma concentrations in TB [38]. This indicates that higher levels of IL-7 do not necessarily imply protection in active TB patients, although it could be used as a potential biomarker of TB.

Other individual serum markers identified in our work included IL-6 and TGFα, both showing a good performance differentiating active TB from uninfected contacts. Our results agree with previous studies [12–14,16] that had reported diagnostic potential of IL-6, which indicates the robustness of this biomarker in different geographical settings. However, we have not found in the literature previous references of TGFα as a potential TB biomarker, which suggests that further research is required to investigate this new candidate.

It is also worth mention that one of the markers included in our study, TNFα, showed a good performance differentiating uninfected and LTBI contacts. This finding has a particular interest, as differentiation between healthy individuals with or without latent infection is normally only possible after antigen-specific activation. TNFα is a critical cytokine for granuloma formation and maintenance, with an important role in controlling *Mtb* infection [39]. This would explain why patients with latent infection have higher levels of TNFα than uninfected contacts.

Regarding the marker signature found in saliva, Fractalkine was the individual marker with the best performance differentiating active TB and NoTBI contacts. A higher concentration of Fractalkine in saliva from active TB patients was also found by Phalane *et al*. [16], supporting our results. In addition, our work also provides information of a higher concentration of this chemokine in LTBI contacts and a good performance differentiating LTBI from uninfected contacts. These findings indicate the interest of this chemokine and its potential role as a saliva TB biomarker.

Our study suggested that discrimination between active TB and NoTBI contacts could be done using Fractalkine alone or in combination with IP-10, IL-1α and VEGF, which provided a higher sensitivity and specificity than any of the markers individually. Three of these candidates, IP-10, Fractalkine and VEGF were found in higher concentrations in saliva from active TB patients, while IL-1α was more abundant in NoTBI patients. Higher concentration of IP-10 could be related with an active cell migration, as discussed above, which could be also reflected in saliva. In the case of Fractalkine and VEGF, we believe there is a connection between their higher concentration in saliva from active TB patients and increased levels of these chemokines in lung cells [40], bronchoalveolar lavage [41] and pleural effusions [42] previously reported in patients with pulmonary tuberculosis. These observations suggest that biomarkers found in saliva could be reflecting the events that take place in the lungs.

Both Fractalkine and VEGF are expressed by endothelial cells to mediate lymphocyte chemoattraction and adhesion in mucosal tissues [43] and promote formation of new blood vessels [44], respectively. Although the role of these proteins during TB infection has not been established, previous studies in animal models suggested that Fractalkine could mediate cellular infection and spread of *Mtb* bacilli [45] and VEGF has been suggested as a mechanism for *Mtb* spreading [46]. All this suggests that endothelial cells have an influence on the markers detected in saliva, and could be indirectly related with *Mtb* spreading in active TB patients.

Besides those included in the four-marker panel, other individual candidate markers were detected in saliva that could discriminate active TB and NoTBI contacts. These included GRO, MCP-1 and MIP-1α, three chemokines known to be involved in the recruitment of neutrophils [47] and in the neutrophil-mediated immune response against *Mtb* infection [48]. In addition, there exist evidences suggesting that GRO could be related to TB pathogenesis in the lungs [49]; MCP-1 is more produced by monocytes from active TB patients and healthy controls [50] and MIP-1α was seen to contribute to the innate immune response to *M*. *tuberculosis* infection [51]. All these evidences suggest that markers detected in saliva samples might reflect the innate-mediated mechanisms triggered against *Mtb* infection.

Our study also indicated that discrimination of LTBI contacts and active TB patients using saliva could be possible using IP-10 or IFNα2. To our knowledge, this is the first time IFNα2 is proposed as a potential biomarker to discriminate active and latent TB infection in saliva samples. On the other hand, for discrimination between uninfected and LTBI contacts, IL-12p40 was the most promising candidate.

IL-12p40 and the already mentioned IL-1α were the only cytokines identified in our study that presented higher concentrations in NoTBI contacts. Higher concentrations of these markers in uninfected contacts could correlate with studies suggesting their role in protection against *Mtb* infection during the first stages of the disease [52–55].

One limitation of our study is its relatively small sample size. However, this was planned as a discovery study to provide new candidate markers that could be further evaluated in future works. Therefore, we prioritized the quality of the study cohort by selecting only those patients and contacts without any condition that could interfere with the immune response signature. Hence allowing for an effective analysis of the immune responses without interferences. With the current analysis, we provided confirmation of previous findings, such as the great potential of IP-10 not only in blood-derived samples, but also in saliva, and we also provided data supporting the potential of IL-6 and Fractalkine as serum and saliva biomarkers, respectively. More importantly, we propose new candidates to be used in serum (TGFα, IL-7) or saliva (MIP1α, VEGF, GRO, MCP-1) to differentiate different TB study groups and biomarker combinations with promising discriminatory capacity that could set the basis for point-of-care tests based on affordable platforms.

Marker combinations proposed for its use in serum samples included IP-10+IL-7 or/and IP-10+BCA-1 for discrimination between active TB patients and uninfected and latently infected contacts, respectively. As for the marker combinations proposed for its use in saliva, Fractalkine + IP-10 + IL-1α + VEGF can discriminate active TB from NoTBI contacts. Finally, the study of Fractalkine + IL-12p40 could differentiate between uninfected and LTBI contacts, being of special interest taking into account that until now, no other methods than the Tuberculin skin test or the Interferon gamma release assays (IGRA) can discriminate between these two populations.

## Supporting information

S1 Table Median levels and interquartile ranges of selected candidate host markers detected in serum samples from TB patients, uninfected and with latent infection contacts and their p values and diagnostic performance. (DOCX)

S2 Table Median levels and interquartile ranges of selected candidate host markers detected in saliva samples from the TB patients, uninfected and with latent infection contacts, and their p values and diagnostic performance. (DOCX)
