# Supplementary material for: Identification of candidate host serum and saliva biomarkers for a better diagnosis of active and latent tuberculosis infection
Source: PLoS One. 2020 Jul 20;15(7):e0235859. doi: 10.1371/journal.pone.0235859 (PMC7371182; doi:10.1371/journal.pone.0235859)
Supplement: S1 Table — (DOCX) [file pone.0235859.s001.docx]

**Table S1**. **Median levels and interquartile ranges of selected candidate host markers detected in serum samples from TB patients, uninfected and with latent infection contacts and their p values and diagnostic performance.**

| ***NoTBI vs. TB - Serum*** | | | | | | | |
| --- | --- | --- | --- | --- | --- | --- | --- |
| **Marker** | **Median (interquartile range)** | | ***p* value** | **AUC** | **Cut off** | **Sensitivity %  (95% CI)** | **Specificity %  (95% CI)** |
|  | **NoTBI** | **TB** |  |  |  |  |  |
| **IL-6** | 0  (0-9.38) | 20  (5.74-45.14) | 0.0009 | **0.7894** | > 9.956 | 70.83  (48.91 - 87.38) | 77.78  (60.85 - 89.88) |
| **IL-7** | 3.00  (0.91-4.78) | 10.12  (5.08-26.36) | 0.0028 | **0.8267** | > 5.312 | 76  (54.87 - 90.64) | **85.71**  (18.11 - 61.56) |
| **IP-10** | 191.6  (157.6-329.9) | 483.5  (309.1-738.6) | < 0.0001 | **0.827** | > 466.5 | 64  (42.52 - 82.03) | **91.89**  (78.09 - 98.30) |
| **TGFα** | 4.73  (3.36-8.62) | 10.84  (7.76-19.38) | 0.0005 | **0.8129** | > 7.218 | **84**  (63.92 - 95.46) | 67.74  (48.63 - 83.32) |
| **TNFα** | 8.74  (6.3-11.57) | 20.32  (15.02-32.79) | < 0.0001 | **0.8519** | > 12.97 | **84**  (63.92 - 95.46) | **81.08**  (64.84 - 92.04) |
| **BCA-1** | 23.26  (15.28-31.26) | 37.4  (25.37-58.73) | 0.0088 | **0.7111** | > 31.96 | 64  (42.52 - 82.03) | 80.56  (63.98 - 91.81) |
| ***LTBI vs. TB - Serum*** | | | | | | | |
| **Marker** | **Median (interquartile range)** | | ***p* value** | **AUC** | **Cut off** | **Sensitivity %  (95% CI)** | **Specificity %  (95% CI)** |
|  | **LTBI** | **TB** |  |  |  |  |  |
| **IL-6** | 2.74  (0-111.5) | 20  (5.74-45.14) | 0.2142 | 0.6181 | > 0.0292 | **100**  (85.75 - 100.0) | 45.83  (25.55 - 67.18) |
| **IL-7** | 3.74  (0.19-12.73) | 10.12  (5.08-26.36) | 0.0656 | **0.7267** | > 5.391 | 76  (54.87 - 90.64) | 66.67  (40.99 - 86.66) |
| **IP-10** | 282  (167.7-352.9) | 483.5  (309.1-738.6) | 0.0059 | **0.776** | > 450.9 | 64  (42.52 - 82.03) | **92**  (73.97 - 99.02) |
| **TGFα** | 5.179  (3.36-20.18) | 10.84  (7.76-19.38) | 0.0394 | 0.6817 | > 6.968 | **84**  (63.92 - 95.46) | 62.5  (40.59 - 81.20) |
| **TNFα** | 16.8  (9.53-42.01) | 20.32  (15.02-32.79) | 0.6511 | 0.5888 | > 12.22 | **88**  (68.78 - 97.45) | 40  (21.13 - 61.33) |
| **BCA-1** | 23.56 (17.15-27.36) | 37.4  (25.37-58.73) | 0.005 | **0.7784** | > 28.03 | 72  (50.61 - 87.93) | **80**  (59.30 - 93.17) |
| ***NoTBI vs. LTBI - Serum*** | | | | | | | |
| **Marker** | **Median (interquartile range)** | | ***p* value** | **AUC** | **Cut off** | **Sensitivity %  (95% CI)** | **Specificity %  (95% CI)** |
|  | **NoTBI** | **LTBI** |  |  |  |  |  |
| **IL-6** | 0  (0-9.38) | 2.74  (0-111.5) | 0.3014 | 0.603 | > 80.56 | 33.33  (15.63 - 55.32) | **97.22**  (85.47 - 99.93) |
| **IL-7** | 3.00  (0.91-4.78) | 3.74  (0.19-12.73) | 0.2728 | 0.5397 | > 5.191 | 38.89  (17.30 - 64.25) | 85.71  (63.66 - 96.95) |
| **IP-10** | 191.6  (157.6-329.9) | 282  (167.7-352.9) | 0.7338 | 0.6022 | > 245.1 | 60  (38.67 - 78.87) | 67.57  (50.21 - 81.99) |
| **TGFα** | 4.73  (3.36-8.62) | 5.179  (3.36-20.18) | 0.7934 | 0.5685 | > 16.46 | 29.17  (12.62 - 51.09) | **96.77**  (83.30 - 99.92) |
| **TNFα** | 8.74  (6.3-11.57) | 16.8  (9.53-42.01) | 0.0039 | **0.7335** | > 14.91 | 60  (38.67 - 78.87) | 83.78  (67.99 - 93.81) |
| **BCA-1** | 23.26  (15.28-31.26) | 23.56  (17.15-27.36) | > 0.999 | 0.5194 | < 37.96 | 96  (79.65 - 99.90) | 19.44  (8.194 - 36.02) |

***TB:*** *Active tuberculosis patients;* ***NoTBI****: Uninfected contacts.* ***LTBI****: Contacts with latent TB infection;* ***AUC****: Area under the ROC curve;* ***Cut off****: marker concentration cut off with the best Youden index;* ***CI****: Confidence interval.*
