# Supplementary material for: Identification of candidate host serum and saliva biomarkers for a better diagnosis of active and latent tuberculosis infection
Source: PLoS One. 2020 Jul 20;15(7):e0235859. doi: 10.1371/journal.pone.0235859 (PMC7371182; doi:10.1371/journal.pone.0235859)
Supplement: S2 Table — (DOCX) [file pone.0235859.s002.docx]

**Table S2.** **Median levels and interquartile ranges of selected candidate host markers detected in saliva samples from the TB patients, uninfected and with latent infection contacts, and their p values and diagnostic performance.**

| ***NoTBI vs. TB- saliva*** | | | | | | | | | | | | | | | | |
| --- | --- | --- | --- | --- | --- | --- | --- | --- | --- | --- | --- | --- | --- | --- | --- | --- |
| **Marker** | **Median (interquartile range)** | | | ***p* value** | **AUC** | | **Cut off** | | | | **Sensitivity %  (95% CI)** | | | **Specificity %  (95% CI)** | | |
|  | **NoTBI** | **TB** | |  |  |  |  |  |  |  |  |  |  |  |  |  |
| **TGFα** | 4.92  (3.20-6.74) | 7.822  (4.53-12.89) | | 0.0241 | 0.6975 | | > 6.978 | | | | 57.14  (37.18 - 75.54) | | | 79.41  (62.10 - 91.30) | | |
| **Fractalkine** | 179.4  (123.7-206.4) | 346.1  (244.4-474.6) | | <0.0001 | **0.8705** | | > 239.3 | | | | 78.57  (59.05 - 91.70) | | | **81.25**  (63.56 - 92.79) | | |
| **IFNα2** | 19.7  (11.15-39.24) | 32.91  (18.1-63.53) | | 0.0315 | 0.6858 | | > 26.85 | | | | 71.43  (51.33 - 86.78) | | | 65.63  (46.81 - 81.43) | | |
| **GRO** | 35.77  (24.87-92.34) | 158.1  (51.72-661.4) | | 0.0011 | **0.7684** | | > 104.0 | | | | 71.43  (51.33 - 86.78) | | | 78.79  (61.09 - 91.02) | | |
| **IL-12p40** | 4.807  (1.76-18.88) | 2.326  (0-5.2) | | 0.0686 | 0.6719 | | < 6.173 | | | | 85.19  (66.27 - 95.81) | | | 46.88  (29.09 - 65.26) | | |
| **IL-1α** | 1331  (726.8-2430) | 670.5  (415.6-840.5) | | 0.0009 | **0.7745** | | < 950.3 | | | | **81.48**  (61.92 - 93.70) | | | 64.71  (46.49 - 80.25) | | |
| **IL-6** | 2.1  (0.11-5.27) | 6.1  (1.68-11.56) | | 0.043 | 0.6863 | | > 4.458 | | | | 65.38  (44.33 - 82.79) | | | 71.88  (53.25 - 86.25) | | |
| **IP-10** | 21.1  (0-57.04) | 72.49  (27.6-415.3) | | 0.0012 | **0.7803** | | > 26.71 | | | | **80.77**  (60.65 - 93.45) | | | 63.64  (45.12 - 79.60) | | |
| **MCP-1** | 140.5  (43.23-285) | 349.8  (152.8-589.3) | | 0.0039 | **0.7416** | | > 312.4 | | | | 60.71  (40.58 - 78.50) | | | **82.35**  (65.47 - 93.24) | | |
| **MIP-1α** | 5.719  (1.6-11.1) | 11.32  (5.766-25.55) | | 0.0085 | **0.734** | | > 3.920 | | | | **96.3**  (81.03 - 99.91) | | | 43.33  (25.46 - 62.57) | | |
| **VEGF** | 48.57  (27.79-104.9) | 124.7  (56.14-260.3) | | 0.0069 | **0.73** | | > 63.17 | | | | 75  (55.13 - 89.31) | | | 66.67  (48.17 - 82.04) | | |
| ***LTBI vs. TB-saliva*** | | | | | | | | | | | | | | | |  |
| **Marker** | **Median (interquartile range)** | | | ***p* value** | | **AUC** | | **Cut off** | | **Sensitivity %  (95% CI)** | | | **Specificity %  (95% CI)** | | |  |
|  | **LTBI** | **TB** | |  | |  | |  | |  | | |  | | |  |
| **TGFα** | 5.56  (3.59-9.18) | 7.822  (4.53-12.89) | | 0.4557 | | 0.6120 | | > 7.876 | | 50  (30.65 - 69.35) | | | 73.08  (52.21 - 88.43) | | |  |
| **Fractalkine** | 258.8  (192.3-381.4) | 346.1  (244.4-474.6) | | 0.1655 | | 0.6573 | | > 338.2 | | 53.57  (33.87 - 72.49) | | | 73.08  (52.21 - 88.43) | | |  |
| **IFNα2** | 24.55  (10.16-31.24) | 32.91  (18.1-63.53) | | 0.0383 | | **0.7098** | | > 26.73 | | 71.43  (51.33 - 86.78) | | | 62.5  (40.59 - 81.20) | | |  |
| **GRO** | 65.38  (25.69-300.6) | 158.1  (51.72-661.4) | | 0.0928 | | 0.6683 | | > 103.5 | | 71.43  (51.33 - 86.78) | | | 57.69  (36.92 - 76.65) | | |  |
| **IL-12p40** | 0  (0-3.756) | 2.326  (0-5.2) | | >0.999 | | 0.5679 | | > 3.787 | | 44.44  (25.48 - 64.67) | | | 77.78  (57.74 - 91.38) | | |  |
| **IL-1α** | 965  (436.2-1559) | 670.5  (415.6-840.5) | | 0.2772 | | 0.6283 | | < 859.2 | | 77.78  (57.74 - 91.38) | | | 59.26  (38.80 - 77.61) | | |  |
| **IL-6** | 2.0  (0.32-5.97) | 6.1  (1.68-11.56) | | 0.0569 | | 0.6939 | | > 5.540 | | 61.54  (40.57 - 79.77) | | | 75  (53.29 - 90.23) | | |  |
| **IP-10** | 13.05  (0-56.35) | 72.49  (27.6-415.3) | | 0.0007 | | **0.7800** | | > 27.45 | | **80.77**  (60.65 - 93.45) | | | 68  (46.50 - 85.05) | | |  |
| **MCP-1** | 141.9  (75.96-647.2) | 349.8  (152.8-589.3) | | 0.2098 | | 0.6376 | | > 146.8 | | **82.14**  (63.11 - 93.94) | | | 51.85  (31.95 - 71.33) | | |  |
| **MIP-1α** | 7.123  (1.851-13.77) | 11.32  (5.766-25.55) | | 0.1479 | | 0.6551 | | > 3.123 | | **100**  (87.23 - 100.0) | | | 29.17  (12.62 - 51.09) | | |  |
| **VEGF** | 60.28  (31.76-126.4) | 124.7  (56.14-260.3) | | 0.0496 | | 0.6845 | | > 85.05 | | 67.86  (47.65 - 84.12) | | | 66.67  (46.04 - 83.48) | | |  |
| ***NoTBI vs. LTBI-saliva*** | | | | | | | | | | | | | | |  |  |
| **Marker** | **Median (interquartile range)** | | | ***p* value** | | **AUC** | | **Cut off** | **Sensitivity %  (95% CI)** | | | **Specificity %  (95% CI)** | | |  |  |
|  | **NoTBI** | | **LTBI** |  | |  | |  |  | | |  | | |  |  |
| **TGFα** | 4.92  (3.20-6.74) | | 5.56  (3.59-9.18) | 0.8153 | | 0.582 | | > 7.018 | 42.31  (23.35 - 63.08) | | | 79.41  (62.10 - 91.30) | | |  |  |
| **Fractalkine** | 179.4  (123.7-206.4) | | 258.8  (192.3-381.4) | 0.0107 | | **0.7284** | | > 205.0 | 69.23  (48.21 - 85.67) | | | 75  (56.59 - 88.54) | | |  |  |
| **IFNα2** | 19.7  (11.15-39.24) | | 24.55  (10.16-31.24) | >0.999 | | 0.6858 | | < 36.87 | 87.5  (67.64 - 97.34) | | | 31.25  (16.12 - 50.01) | | |  |  |
| **GRO** | 35.77  (24.87-92.34) | | 65.38  (25.69-300.6) | 0.6242 | | 0.5938 | | > 68.43 | 50  (29.93 - 70.07) | | | 72.73  (54.48 - 86.70) | | |  |  |
| **IL-12p40** | 4.807  (1.76-18.88) | | 0  (0-3.756) | 0.0052 | | **0.7280** | | < 2.887 | 74.07  (53.71 - 88.89) | | | 71.88  (53.25 - 86.25) | | |  |  |
| **IL-1α** | 1331  (726.8-2430) | | 965  (436.2-1559) | 0.1972 | | 0.6340 | | < 2040 | 88.89  (70.84 - 97.65) | | | 38.24  (22.17 - 56.44) | | |  |  |
| **IL-6** | 2.1  (0.11-5.27) | | 2.0  (0.32-5.97) | >0.999 | | 0.5039 | | < 0.365 | 37.5  (18.80 - 59.41) | | | 71.88  (53.25 - 86.25) | | |  |  |
| **IP-10** | 21.1  (0-57.04) | | 13.05  (0-56.35) | >0.999 | | 0.5436 | | < 23.05 | 64  (42.52 - 82.03) | | | 48.48  (30.80 - 66.46) | | |  |  |
| **MCP-1** | 140.5  (43.23-285) | | 141.9  (75.96-647.2) | 0.5961 | | 0.5926 | | > 73.61 | 81.48  (61.92 - 93.70) | | | 41.18  (24.65 - 59.30) | | |  |  |
| **MIP-1α** | 5.719  (1.6-11.1) | | 7.123  (1.851-13.77) | >0.999 | | 0.5653 | | > 5.099 | 70.83  (48.91 - 87.38) | | | 50  (31.30 - 68.70) | | |  |  |
| **VEGF** | 48.57  (27.79-104.9) | | 60.28  (31.76-126.4) | >0.999 | | 0.5370 | | > 59.20 | 51.85  (31.95 - 71.33) | | | 66.67  (48.17 - 82.04) | | |  |  |

***TB****: Active Tuberculosis patients;* ***LTBI****: Contacts with latent infection;* ***NoTBI****: Uninfected contacts;* ***AUC****: Area under the ROC curve;* ***Cut off****: marker concentration cut off with the best Youden index;* ***CI****: Confidence interval.*
